# Molecular Characterization of Newcastle Disease Viruses Isolated from Chickens in Tanzania and Ghana

**DOI:** 10.3390/v12090916

**Published:** 2020-08-20

**Authors:** Ana P. da Silva, Emily J. Aston, Gaspar H. Chiwanga, Ashley Birakos, Amandus P. Muhairwa, Boniface B. Kayang, Terra Kelly, Huaijun Zhou, Rodrigo A. Gallardo

**Affiliations:** 1 Department of Population Health and Reproduction, School of Veterinary Medicine, University of California, Davis, CA 95616, USA; apdasilva@ucdavis.edu (A.P.d.S.); akbirakos@ucdavis.edu (A.B.); 2 Department of Animal Science, College of Agricultural and Environmental Sciences, University of California, Davis, CA 95616, USA; ejaston@ucdavis.edu (E.J.A.); hzhou@ucdavis.edu (H.Z.); 3 Department of Veterinary Medicine and Public Health, College of Veterinary Medicine and Biomedical Sciences, Sokoine University of Agriculture, Morogoro, Tanzania; chiwanga2000@yahoo.com (G.H.C.); apm@sua.ac.tz (A.P.M.); 4 Department of Animal Science, University of Ghana, Legon, Accra 233, Ghana; bbkayang@ug.edu.gh; 5 One Health Institute, School of Veterinary Medicine, University of California, Davis, CA 95616, USA; trkelly@ucdavis.edu

**Keywords:** Newcastle disease, molecular characterization, Africa, MinION, Nanopore, poultry

## Abstract

Newcastle disease (ND) is one of the most challenging infectious diseases affecting poultry production in Africa, causing major economic losses. To date, Newcastle disease virus isolates from several African countries have been grouped into class II NDV genotypes I, IV, V, VI, VII, XI, XIII, XIV, XVII, XVIII and XXI. Although ND is endemic in many African countries, information on circulating genotypes is still scarce. In Tanzania, outbreaks with genotypes V and XIII have been reported. In West and Central Africa, genotypes XIV, XVII, and XVIII are the most predominant. To investigate other genotypes circulating in Tanzania and Ghana, we performed molecular genotyping on isolates from Tanzania and Ghana using the MinION, a third-generation portable sequencing device from Oxford Nanopore Technologies. Using the MinION, we successfully sequenced the NDV F gene hypervariable region of 24 isolates from Tanzania and four samples from Ghana. In Tanzania, genotypes V, VII and XIII were detected. All isolates from Ghana belonged to genotype XVIII. The data obtained in this study reflect the genetic diversity of NDV in Africa and highlight the importance of surveillance for monitoring the distribution of NDV genotypes and viral evolution.

## 1. Introduction

Newcastle disease (ND) is a highly transmissible viral disease that affects many avian species, including domestic poultry [1]. The causative agent, Newcastle disease virus (NDV), is a paramyxovirus classified as avian orthoavulavirus 1, previously known as avian paramyxovirus 1 (APMV-1) [2]. NDV is an enveloped, negative sense, single-stranded RNA virus [3]. The viral envelope is composed of two glycoproteins—the hemagglutinin-neuraminidase (HN) and the fusion (F) proteins. In addition to inducing hemagglutination of red blood cells, the HN protein is responsible for the virus binding to host cell receptors, which is facilitated by its neuraminidase activity. In addition, the HN protein activates the F protein, which in turn induces the fusion of the viral envelope to the cell membrane [3]. Because of its high molecular variability and association with virulence, the F gene has been widely used as a target for the genotypic classification of NDV [4,5]. Based on the complete sequence of the F gene, NDV strains are divided into two major groups—class I and class II. Class I viruses belong to a single genotype, whereas the more genetically diverse class II viruses are currently divided into 20 distinct genotypes separated by nucleotide distances above 10% [5]. Partial F gene sequences have also been used successfully to genotype NDV isolates [6,7].

ND is caused by infection with virulent (v)NDV strains. Clinical signs can range from respiratory distress to neurologic signs and severe systemic illness, and mortality can reach 100%. Depending on the clinical signs observed in chickens after experimental inoculation, strains can be divided into lentogenic, mesogenic or velogenic (listed in increasing order of virulence) [1]. NDV strains are considered velogenic when they have an intracerebral pathogenicity index (ICPI) in day-old chicks of 0.7 or greater or contain a cleavage site with multiple basic amino acids at the C-terminus of the F2 protein and phenylalanine at the N-terminus of the F1 protein (residue 117) [1,8].

Outbreaks of vNDV occur worldwide and the disease is endemic in many countries in Asia, the Middle East and Africa [1,9]. In Africa, local poultry production largely consists of free-range indigenous chickens [10], many of which are multi-age flocks that have not been vaccinated against NDV. These chickens may also originate from different geographic locations, and some are sold in live bird markets, where the birds are housed in close proximity and with other avian species. These factors facilitate the circulation and dissemination of various vNDV genotypes, which complicate disease prevention and control measures in these countries [11,12,13,14,15]. To date, NDV isolates from several African countries have been grouped into genotypes I, IV, V, VI, VII, XI, XIII, XIV, XVII, XVIII and XXI, according to the recently updated NDV classification system [5]. In a 2012 study in Tanzania, NDV isolates from chickens in live bird markets representing six different regions were classified as genotype V and XIII [15]. In West and Central Africa, NDV samples belonging to genotypes XIV, XVII, and XVIII were collected between 2006 and 2011 from poultry in live bird markets and backyard and commercial farms [14].

NDV genomic surveillance is essential for providing insights on genetic variability in circulating strains and for improving preparedness and response to disease outbreaks. In addition, up-to-date genotype investigations are essential to determine geographic distributions of genotypes and maintain and improve current molecular classification methods [5]. The evolution of sequencing technologies has greatly upgraded genomics as a whole [16]. In molecular virology, third-generation sequencing has revolutionized the ability to sequence complete viral genomes at depth rapidly and with high accuracy [7,17,18]. Of the many high-throughput platforms available, the MinION (Oxford Nanopore Technologies, Oxford, UK) is particularly valuable due to its low cost, easy set-up and portability, which enables it to be deployed in remote locations where sequencing services are inaccessible.

Although ND is endemic in many African countries, information on circulating genotypes is still scarce. In this study, a protocol using the MinION was developed to investigate NDV genotypes circulating in indigenous chickens in Tanzania and Ghana.

## 2. Materials and Methods

### 2.1. Isolates from Tanzania and Samples from Ghana

The vNDV isolates from Tanzania and the NDV-positive swab samples from Ghana used in this study are part of previous and independent projects and considered archived samples at the Sokoine University of Agriculture and University of Ghana poultry medicine laboratories.

**Figure 1:**
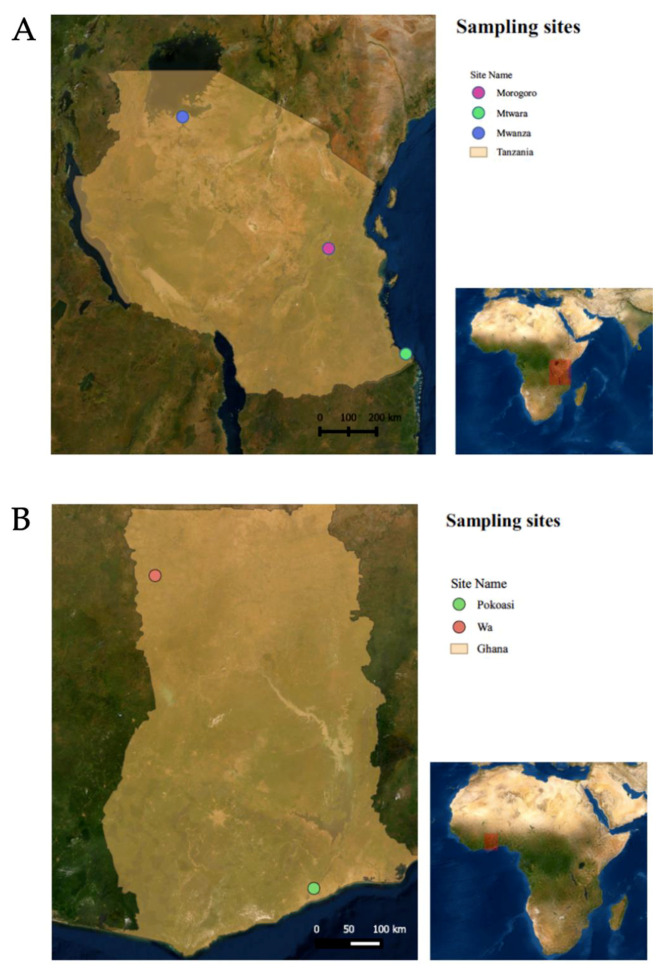
Sample sites for (**A**) Tanzania (Morogoro, Mtwara and Mwanza) and (**B**) Ghana (Pokoasi and Wa).

Twenty-nine vNDV strains from Tanzania were isolated in 9- to 11-day-old SPF embryonated eggs as previously described [19]. The isolates originated from sick chickens diagnosed between 2011 and 2017. Of the 29 isolates, the partial F gene was successfully amplified in 25 samples, and 24 samples were selected for sequencing. In Ghana, RT-PCR amplification was attempted from oropharyngeal/cloacal swabs, without previous isolation in eggs, from 27 clinically ill chickens in 2018. Of these, the partial F gene was successfully amplified from four swabs by RT-PCR and subsequent sequencing. The 29 isolates from Tanzania and the 27 samples from Ghana had previously tested positive for NDV by a screening RT-qPCR assay [20]. The sample collection locations are presented in Figure 1.

### 2.2. Partial F Gene Amplification

RNA extraction from allantoic fluid or oropharyngeal/cloacal swabs was performed using the MagMAX Viral/Pathogen Nucleic Acid Isolation Kit (Thermo Fisher Scientific, Waltham, MA, USA) following the manufacturer’s instructions. A conventional RT-PCR assay was performed using the QIAgen OneStep RT-PCR Kit (QIAgen, Valencia, CA, USA). Primers 4331F and 5090R were used to amplify a 788-bp fragment that includes the 3′ end of the M gene (173 bp) and the 5′ end of the F gene, which comprises the F gene hypervariable region (615 bp) [6,7]. The amplicons were separated by electrophoresis in a 2% agarose gel, stained with GelGreen (Phenix Research, Candler, NC, USA) and visualized under ultraviolet light.

### 2.3. Library Preparation and Sequencing

RT-PCR products were purified using the Agencourt AMPure XP beads (Beckman Coulter, Indianapolis IN). The amplicons were barcoded using the Native Barcoding Expansion Kit (Oxford Nanopore Technologies, Oxford, UK). Samples were pooled and the DNA library preparation was performed using the Ligation Sequencing Kit (Oxford Nanopore Technologies, Oxford, UK). A maximum of 12 samples were barcoded and pooled per library prep. Sequencing was performed on a MinION (Oxford Nanopore Technologies) and ran for at least 12 h; two runs were performed in Tanzania (12 barcoded samples each) and one in Ghana (four barcoded samples). Reads were demultiplexed using the Nanopore EPI2ME Agent (Oxford Nanopore Technologies). The sequencing data for each sample were assembled using the NDV LaSota strain as a reference (accession number AF077761). For accuracy verification, a second round of reference-based assembly was performed using the consensus sequences obtained with the assembly using LaSota as a reference strain. Sequences were analyzed with the Geneious Prime 2020.1.1 software (https://www.geneious.com).

### 2.4. Phylogenetic Analysis

**Figure 2:**
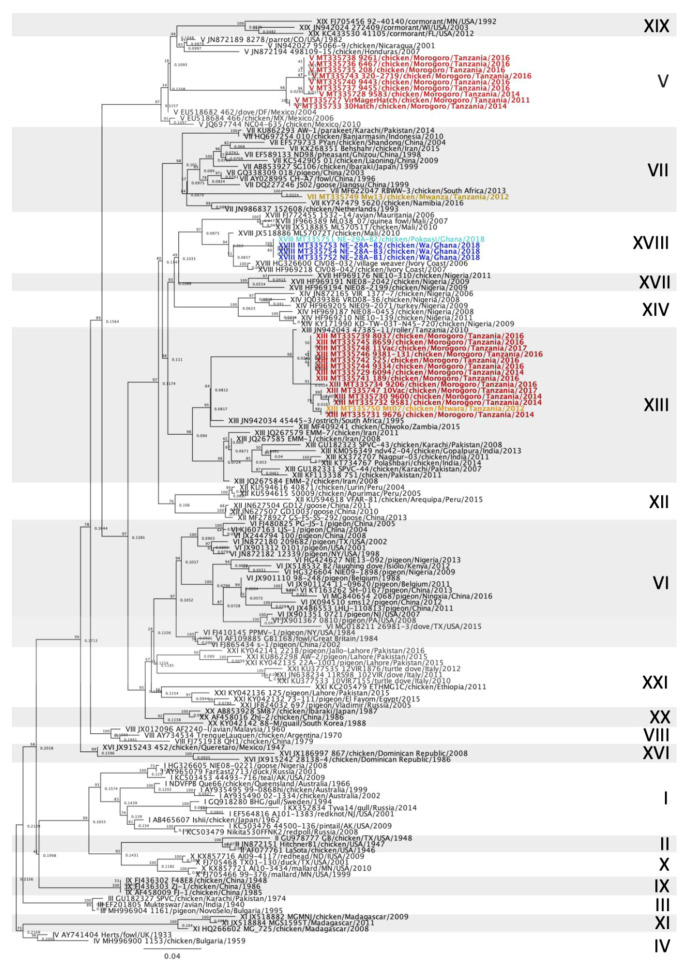
Phylogenetic analysis of Newcastle disease virus class II isolates using a 615-bp fragment of the fusion gene with the maximum likelihood method 1000 bootstrap replicates. Of the 153 sequences used, 28 were collected from diseased chickens from Tanzania and Ghana (bolded and colored) and 125 represent all class II genotypes as described by Dimitrov et al. [5]. The Roman numerals on the right represent the genotypes the isolates belong to. The colors represent the geographic location of the isolates (orange = Mtwara, red = Morogoro, yellow = Mwanza, light blue = Pokoasi and dark blue = Wa).

**Figure 3:**
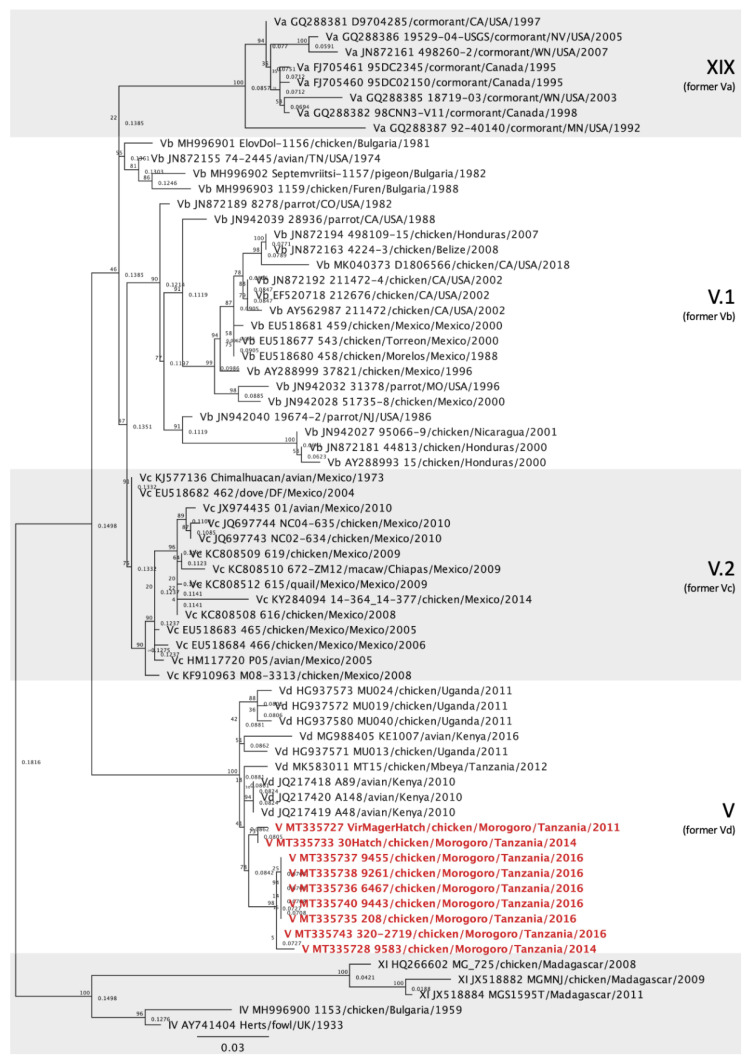
Phylogenetic analysis of Newcastle disease virus genotype V isolates using a 615-bp fragment of the fusion gene with the maximum likelihood method with 1000 bootstrap replicates. Of the 67 sequences used, 9 were collected from diseased chickens from Morogoro, Tanzania (bolded and colored), 53 represent genotype V and XIX isolates [5] and 5 are outliers (2 from genotype IV and 3 from genotype XI). The Roman numerals on the right represent the subgenotypes the isolates belong to. The previous terminology [4] is listed below the current nomenclature in parenthesis for reference.

**Figure 4:**
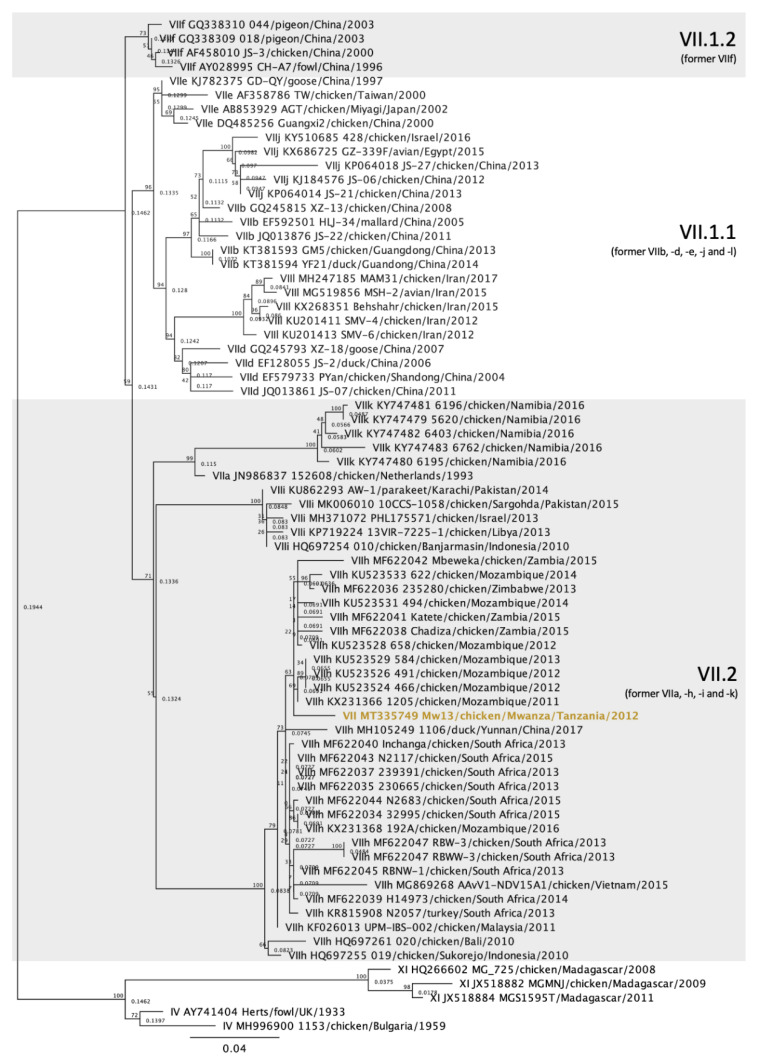
Phylogenetic analysis of Newcastle disease virus genotype VII isolates using a 615-bp fragment of the fusion gene with the maximum likelihood method with 1000 bootstrap replicates. Of the 72 sequences used, one was collected from diseased chickens from Mwanza, Tanzania (bolded and colored), 66 represent other genotype VII isolates [5], and 5 are outliers (2 from genotype IV and 3 from genotype XI). The Roman numerals on the right represent the subgenotypes the isolates belong to. The previous terminology [4] is listed below the current nomenclature in parenthesis for reference.

**Figure 5:**
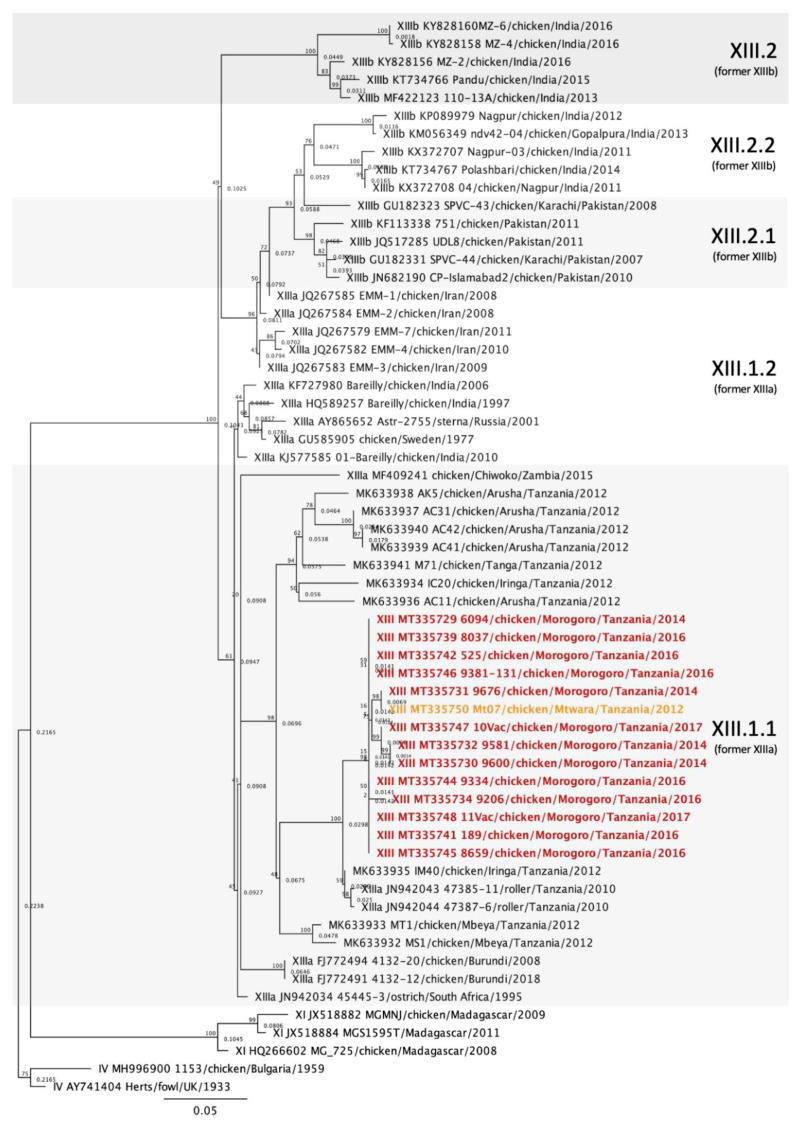
Phylogenetic analysis of Newcastle disease virus genotype XIII isolates using a 615-bp fragment of the fusion gene with the maximum likelihood method with 1000 bootstrap replicates. Of the 60 sequences used, 13 were collected from diseased chickens from Morogoro and one from Mtwara, Tanzania (bolded and colored), 41 represent other genotype XIII isolates [5], and 5 are outliers (2 from genotype IV and 3 from genotype XI). The roman numerals on the right represent the genotypes the isolates belong to. The previous terminology [4] is listed below the current nomenclature in parenthesis for reference. The colors represent the geographic location of the isolates (orange = Mtwara, red = Morogoro).

**Figure 6:**
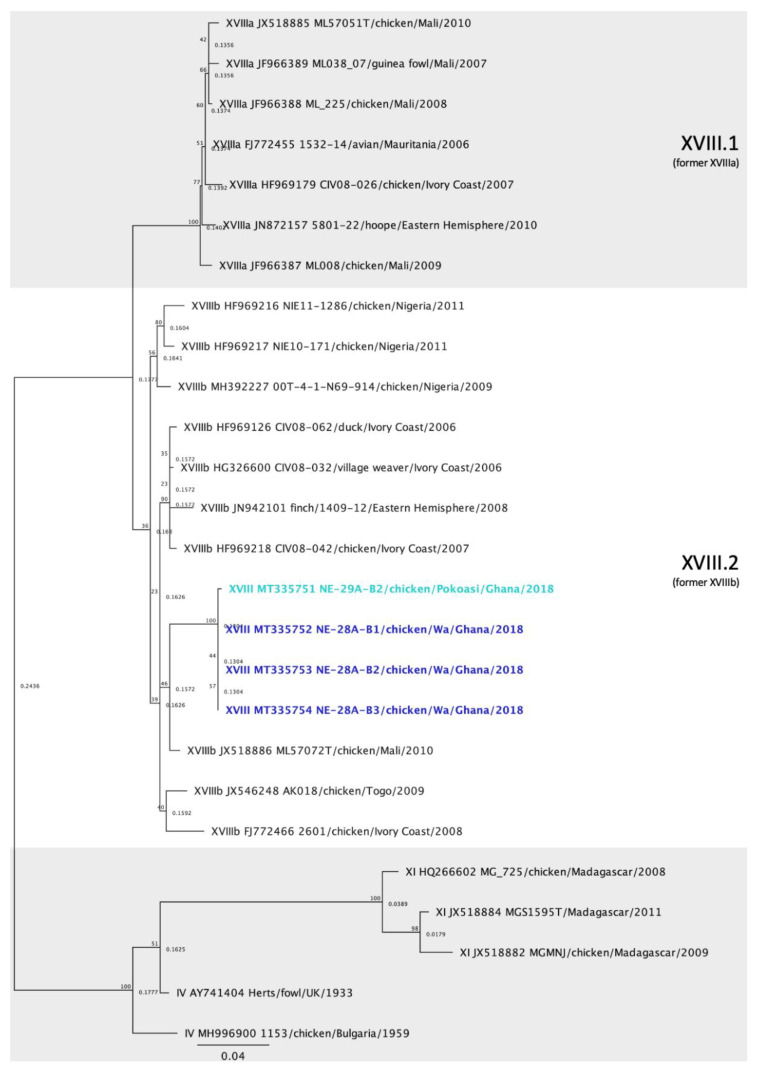
Phylogenetic analysis of Newcastle disease virus genotype XVIII isolates using a 615-bp fragment of the fusion gene with the maximum likelihood method with 1000 bootstrap replicates. Of the 26 sequences used, 3 were collected from diseased chickens from Wa and one from Pokoasi, Ghana (bolded and colored), 17 represent other genotype XVIII isolates [5], and 5 are outliers (2 from genotype IV and 3 from genotype XI). The Roman numerals on the right represent the genotypes the isolates belong to. The previous terminology [4] is listed below the current nomenclature in parenthesis for reference. The colors represent the geographic location of the isolates (light blue = Pokoasi and dark blue = Wa).

The 28 vNDV sequences from Tanzania and Ghana were compared with other NDV sequences deposited in GenBank using the Basic Local Alignment Search Tool (BLAST) and also with isolates of known genotypes [5]. Multiple sequence alignments of the NDV partial F gene were produced in Geneious Prime 2020.1.1 using the MAFFT plugin [21]. The maximum-likelihood method was used to construct phylogenetic trees based on the GTRGAMMAI model with 1000 bootstrap replicates in Geneious Prime using the RaxML plugin [22]. The accession numbers for the Tanzania and Ghana sequences are MT335727 to MT335754. In the initial analysis, the sequences were compared with sequences from 125 other isolates representing all class II ND genotypes [5], totaling 153 sequences (Figure 2). For each genotype detected (V, VII, XIII and XVIII), individual phylogenetic trees were constructed using the most similar sequences detected in BLAST and other relevant isolates detected in Africa (Figure 3, Figure 4, Figure 5 and Figure 6).

## 3. Results

### 3.1. Geographic Distribution of the Sequences

The Tanzania isolates were obtained from chickens presenting with clinical signs of ND in Morogoro (22/24, 91.67%), Mwanza (1/24, 4.27%) and Mtwara (1/24, 4.27%) (Figure 1). From the 22 Morogoro isolates, 18 (81.82%) were obtained from indigenous non-commercial poultry isolated between 2014 and 2016. Two isolates (9.09%) were from breeder flocks in which the disease slowly spread and eventually led to 100% mortality in 2011 and 2014. Two isolates (9.09%) originated from commercial layer flocks that became infected despite being vaccinated against NDV in 2017. The isolates from Mwanza and Mtwara (one from each location) originated from commercial layer hens that were submitted to a veterinary diagnostic laboratory in 2012.

The Ghana sequences originated from oropharyngeal/cloacal swabs collected from clinically ill birds in 2018. One chicken (25%) originated from Pokuasi and three chickens were from Wa (75%) (Figure 1).

### 3.2. Newcastle Disease Virus Pilot Tree

The MinION third generation sequencing strategy allowed the amplification of the F gene hypervariable region of all 28 isolates and samples, yielding at least 2000 reads per sample. Using a smaller dataset of class II sequences retrieved from GenBank, a pilot tree was constructed for rapid preliminary identification of genotypes as previously described [5]. Based on the pilot tree, 14/28 isolates (50%) belonged to genotype XIII, 9/28 sequences (32.14%) to genotype V, 4/28 (14.29%) to genotype XVIII and 1/28 (3.57%) to genotype VII (Figure 2). Based on the OIE definition for vNDV molecular pathotyping [8], all 28 sequences from this study presented at least three basic amino acids at the C-terminus of the F2 protein from residues 112-116 and a phenylalanine at residue 117; therefore, they are all considered virulent strains (Table S1).

### 3.3. Genotype V Isolates

All NDV genotype V isolates were derived from chickens located in Morogoro, Tanzania. Phylogenetic analysis revealed that all nine isolates belong to subgenotype V [5], formerly denominated subgenotype Vd [4] (Figure 3). The Morogoro genotype V isolates shared 85.48 to 100% nucleotide identities. Of these, two (MT335727 and MT335733) were isolated from chickens that originated from the same breeder farm in 2011 and 2014 and were only 93.65% identical. Four genotype V isolates (MT335735, -36, -38 and -40) originated from chickens experimentally exposed to vNDV and were 100% identical. Altogether, the genotype V isolates from Morogoro were closely related to sequences from Mbeya (Tanzania, MK583011) [23], Kenya (JQ217418 to JQ217420) and Uganda (HG937571 to HG937573 and HG937580) [12]. The identities of these genotype V isolates are displayed in Table S2.

### 3.4. Genotype VII Isolate

The single NDV genotype VII isolate was detected in Mwanza, Tanzania, and currently belongs to subgenotype VII.2, previously classified as genotype VIIh (Figure 4) [4,5]. The highest homology of this isolate is to a strain from Mozambique isolated in 2011 (KX231366) [24], sharing 97.87% nucleotide identity. It shares lower identities with sequences from South Africa and Mozambique [24,25], with nucleotide identities ranging from 95.21 to 97.7%. The subgenotype VII.2 identities of the isolates displayed in Figure 4 are in Table S2.

### 3.5. Genotype XIII Isolates

Of the 14 genotype XIII isolates detected in Tanzania, 13 were from Morogoro and one was from Mtwara. All of the isolates clustered together with strains that belonged to the subgenotype XIII.1.1, which were previously denominated XIIIa (Figure 5) [4,5]. Among the 14 isolates, the nucleotide identities varied between 92.49 and 100%, with an average of 98.35% identity. The sequences most similar to the genotype XIII isolates originated from Tanzania (JN942043, JN942044 and MK633935), to which the nucleotide identity averaged 97.37%. Other African subgenotype XIII.1.1 sequences from South Africa (JN942034), Burundi (FJ772491 and FJ772494) and Zambia (MF409241) shared 85.21 to 93.69% homologies with the genotype XIII isolates from this study. The subgenotype XIII.1.1 identities of the isolates displayed in Figure 5 are in Table S2.

### 3.6. Genotype XVIII Isolates

All genotype XVIII isolates were derived from Ghana, with three detected in Wa and one originating from Pokoasi. The four isolates belong to subgenotype XVIII.2, formerly known as XVIIIb (Figure 6) [4,5]. The nucleotide identities between the four isolates ranged from 97.72 to 99.82%. The genotype XVIII sequences were most similar to an isolate from Mali (JX518886), to which they shared between 95.23 to 96.61% nucleotide identity. The subgenotype XVIII.2 identities of the isolates displayed in Figure 6 are in Table S2.

## 4. Discussion

In Africa, ND continues to be a major limitation of poultry production, both in commercial and village poultry production systems, which are important sources of income and food security for many communities [10]. Surveillance efforts to detect circulating NDV genotypes are critical for studying the molecular evolution and spread of NDV in the world. Particularly in Africa, where ND is endemic, understanding NDV diversity and distribution is valuable in recognizing disease patterns, investigating relationships between isolates in different geographical locations to evaluate spread and predicting outbreaks, and for informing on targeted biosecurity measures, including vaccine development and vaccination strategies. In this study, the MinION (Oxford Nanopore Technologies, Oxford, UK), a portable sequencing device, was used for onsite sequencing. In addition to providing real-time sequencing with high output data in a matter of hours, the MinION allowed us to perform sequencing in Tanzania and Ghana in laboratories with minimal infrastructure and limited resources, demonstrating its utility in remote locations. Due to restricted access to equipment to assess RNA quality and quantification, sequencing was performed from RT-PCR amplicons focusing on a partial fragment of the F gene as opposed to the full F gene amplification. Although the F gene fragment used in the study contains the F gene hypervariable region, the full-length F gene is preferred for molecular characterization [5].

Of the 28 sequences obtained in this study, 24 originated from Tanzania and 4 from Ghana. In Tanzania, the partial F gene amplification by RT-PCR was successful in 25 of the 29 isolates (86.21%), while only 4 of the 27 swab samples from Ghana (4.81%) were successfully amplified. In Tanzania, the RT-PCR was performed from NDV strains passaged in SPF eggs, which likely amplified the viral load per µL of allantoic fluid at every passage. In Ghana, the RT-PCR was performed directly from oropharyngeal/cloacal swab samples, which might have reflected a lower viral load and the presence of impurities and PCR inhibitors that might have led to a negative result by conventional RT-PCR. Although RNA samples were stored at −80 °C until processing, it is possible that RNA degradation occurred over time, due to unreliable power supply at times.

Phylogenetic analysis showed that NDV isolates detected in Tanzania belonged to class II genotypes V, VII and XIII, and isolates from Ghana belonged to genotype XVIII (Figure 2). All nine genotype V isolates clustered together and with other sequences that were previously denominated subgenotype Vd [4] (Figure 3). However, according to the most recent NDV phylogenetic classification using the full-length F gene, these sequences lack branch support and are no longer classified into a separate subgenotype [5]. Genotype V isolates have been previously reported in Tanzania [11,15,23], but most of the Tanzanian sequences deposited in GenBank were significantly shorter than the sequences from this study and therefore were not used. The single strain from Tanzania used in this study is from Mbeya [23], which is approximately 630 km southwest of Morogoro, near the northeast region of Zambia. Sequences from Uganda and Kenya, countries bordering Northern Tanzania, also clustered with the isolates from Morogoro. Similar genotype V strains seem to have been circulating within these three East African countries and it has been suggested that uncontrolled cross-border trading of live birds might be responsible for these endemic strains [12,15].

In this study, a single isolate from Tanzania was classified as genotype VII (Figure 2 and Figure 4). To the authors’ knowledge, genotype VII has not yet been reported in Tanzania. This NDV strain was isolated from a commercial laying flock located in the Mwanza lake region, in Northern Tanzania. Genotype VII strains are the most diverse among class II genotypes and have been responsible for widespread outbreaks in Asia, Africa and Europe [26]. Initially, genotype VII sequences were classified into eight subgenotypes (VIIa to -h) [4]. However, with the most recent classification, only three subgenotypes were identified based on nucleotide distances, branch support and number of independent isolates considering the complete F gene [5]. The genotype VII sequence from this study clustered with isolates from subgenotype VII.2, more specifically with the previously known subgenotype VIIh isolates from Mozambique, Zimbabwe, Zambia, South Africa and Asia (Malaysia, China, Indonesia and Vietnam) (Figure 4). The most closely related isolates originated from Mozambique, in which an average of 97.55% nucleotide identity was observed, although nucleotide homology was above 95% with all subgenotype VIIh sequences used in this study (Supplemental Table S2). The distances between the genotype VII sequence detected in this study and other genotype VII from other African countries are likely due to missing data and lack of surveillance between isolation years and between outbreaks. Notably, all subgenotype VIIh isolates from Africa were derived from neighboring countries (South Africa, Zimbabwe, Mozambique, Zambia and Tanzania, Figure 1), which might explain the spread of genotype VIIh isolates within the southeastern region of Africa. The first detection of a genotype VII isolate in Tanzania corroborates the need for continued molecular surveillance of NDV to monitor the evolution and distribution of endemic strains in the continent. Further molecular characterization of this isolate is ultimately necessary for a more in-depth analysis of phylogenetic relationships using full F gene sequences.

The majority of the sequences from this study belong to genotype XIII. In total, 14 genotype XIII isolates were obtained from Tanzania. The nucleotide homologies among the isolates from this study were between 92.49 and 100%, most likely representing different variants of the same strain. With the exception of one strain from Mtwara, all genotype XIII sequences from this study originated from Morogoro, which could justify the genetic homogeneity observed within these isolates. Conversely, a recent study characterizing NDV strains from live bird markets in Tanzania detected nucleotide variability of up to 6% within genotype XIII sequences from Mbeya, Iringa, Arusha and Tanga [15]. This variability is likely due to obtaining the NDV samples in different geographic locations and the origin of the isolates (i.e., from live bird markets). According to the current characterization method, these sequences, as well as the previously mentioned genotype XIII sequences from Tanzania [15], belong to subgenotype XIII1.1 [5]. This subgenotype also includes isolates from Zambia, Burundi and South Africa (Figure 5). The highest nucleotide identity to the sequences presented here occurs with Tanzanian strains JN942043 and JN942044; unfortunately, metadata for these sequences are not available. MK633935, which was isolated from Iringa, presented an average identity of 97.61% to the genotype XIII isolates detected in this study. Remarkably, another subgenotype XIII1.1 isolate from Iringa (MK633934) shared relatively low nucleotide identity to the sequences presented here (87.25–93.51%), suggesting that there might be genotype XIII viruses independently evolving in the live bird markets in Iringa [15].

The sequences obtained from Ghana (*n* = 4) belonged to genotype XVIII, of which three were from Wa and one was from Pokoasi (Figure 2 and Figure 6). Genotype XVIII is composed of strains that circulate in West Africa and have been detected in Mauritania, Mali, Côte d’Ivoire, Togo, Benin and Nigeria [14]. The sequences obtained from Ghana belong to subgenotype XVIII.2 [5], previously denominated XVIIIb [14]. Despite the geographic distance between Wa and Pokoasi (approximately 675 km), the sequences share 97.72 to 99.82% nucleotide homology between each other and are likely variants from the same strain. The sequences from this study branch together and diverge from other subgenotype XVIIIb isolates by approximately 5% nucleotide differences (3.40–7.01%), which might indicate that strains from Ghana are independently evolving from other subgenotype XVIIIb isolates from West Africa. However, molecular characterization using the full-length F gene is required to further assess these relationships.

## 5. Conclusions

The 24 NDV isolates from Tanzania were classified as genotypes V (*n* = 8), VII, (*n* = 1) and XIII (*n* = 15), while the isolates from Ghana were classified as genotype XVIII (*n* = 4). Altogether, the data provide an insight on the circulating NDV genotypes in Tanzania and Ghana and bring awareness to the emergence of genotype VII isolates in Tanzania. Four different genotypes were identified, which reflects the extensive diversity of NDV isolates and variants in the African continent. Integrating pathogen genomics in the surveillance of NDV provides valuable information on virus evolution and the geographical spread of NDV genotypes, highlighting the role of local and international animal trade and movement in the dissemination of ND.

## Supplementary Materials

The following are available online at https://www.mdpi.com/1999-4915/12/9/916/s1. Table S1: Amino acid sequences of the cleavage site of the F gene (residues 112–117) of sequences that belong to Newcastle disease virus genotypes V, VII, XIII and XVIII detected in Tanzania and Ghana. Table S2: Percent nucleotide identity matrices of sequences that belong to Newcastle disease virus genotypes V, VII, XIII and XVIII used in Figure 2, Figure 3, Figure 4, Figure 5 and Figure 6. The identities are based on a 615-bp fragment of the fusion gene of Newcastle disease virus using the MAFFT alignment method.
